# Clinical Trial of Ampicillin in the Treatment of Salmonella Enteritis

**Published:** 1964-10

**Authors:** P. G. Bisson

**Affiliations:** Ham Green Hospital, Bristol


### CLINICAL TRIAL OF AMPICILLIN IN THE TREATMENT OF SALMONELLA ENTERITIS

BY

P. G. BISSON, M.B., CH.B.

*Ham Green Hospital, Bristol*

In the Bristol area bowel infection due to organisms of *Salmonella* species is endemic. For many years various antibiotics, and combinations of antibiotics, have been used in attempts to remove these food poisoning organisms from the stools; the results have been disappointing. When ampicillin (alpha-amino-benzyl penicillin, "Penbritin") became available, it was reported (Rolinson *et al.*, 1961; Rose *et al.*, 1962) that this antibiotic would inhibit the growth *in vitro* of *Salmonella* species, and therefore better results might be expected. A clinical trial was started. At that time a combination of paromomycin ("Humatin") and streptomycin was in use (McMath *et al.*, 1959), this being reputed the best treatment then available. The effect of ampicillin was, therefore, compared with this combination. All patients in the series suffered from infection by *Salmonella heidelberg*.

Patients were either admitted in the acute phase with diarrhoea and vomiting, or were transferred as carriers from other hospitals or nurseries where the organism had been identified in stool cultures. Alternate patients were given either a five day course of ampicillin, 30 mg/kg body weight, or paromomycin, 30 mg/kg body weight per day plus oral streptomycin 1 G twice daily. Treatment was commenced as soon as stools had been sent for culture in order to keep stay in hospital as short as possible, since stool cultures take several days for completion. Patients were considered free from infection if two consecutive stool cultures, taken within fourteen days after treatment, were negative. This test of cure is admittedly modest in its requirement.

### RESULTS WITH AMPICILLIN

Twenty-three patients were treated with this drug. The average length of stay in hospital was twenty-seven days, and the age range was 5–75 years. Thirteen per cent were apparently cleared of infection.

Reactions were experienced by three patients, who developed a macular rash on the face, neck, and trunk, and one also suffered from glossitis. Two of these patients had previously received crystalline penicillin intramuscularly without ill effects.

### RESULTS WITH PAROMOMYCIN AND STREPTOMYCIN

Twenty-four patients were treated with this combination of drugs. The average length of stay in hospital was twenty-seven days and the age ranged from 6 days to 89 years. Of these twenty-four patients fifty per cent were apparently cleared of infection.

### BACTERIOLOGY

Bacteriology was carried out by the Public Health Laboratory Service Laboratory in Bristol. *In vitro*, the ampicillin-sensitivity of the organism isolated from stools after ampicillin treatment was found to be the same as on primary isolation. The sensitivity discs used contained 25 $\mu\mu$g of ampicillin.

### DISCUSSION AND CONCLUSIONS

The *in vitro* activity of ampicillin is not necessarily an index of its therapeutic efficacy; the drug may not reach the organism in the bowel in an active form. Anomalies of this sort are well recognized. However, because such a poor clinical response was obtained with a drug known to be active *in vitro* against *Salmonellae*, a further limited trial was started in which a double dose of ampicillin was used (60 mg/kg body weight per day). After fifteen consecutive patients had been treated with only two successful results the trial was discontinued, as the larger dosage had clearly no greater effect than the smaller.

Recently Sleet *et al.* (1964) obtained encouraging clinical results with ampicillin in paratyphoid infections, using large doses (200 mg/kg/day). Reactions occurred in their patients too, but they considered these of less moment than the more dangerous effects of chloramphenicol. However, in the present trial infections with *Salmonella heidelberg* responded more satisfactorily to paromomycin and streptomycin than to ampicillin in comparable dosage. Perhaps we should be cautious in using ampicillin since the results are poor and the drug is so expensive.

*Acknowledgements*

My thanks are due to Dr. J. Macrae for his help in the preparation of this paper.

### REFERENCES

McMath, W. F. T. and Hussain, K. K. (1959) *Public Health*, **73**, 328.
Rolinson, G. N. and Stevens, S. (1961), *Brit. med. J.* **ii**, 191.
Ross, G., Linein, E. W., Saremba, E. A., Bourjois, L. and Puig, J. R. (1962), *J. Amer. Med. Ass.* **182**, 238.
Sleet, R. A., Sangster, G., and McMurdock, J. (1964), *Brit. med. J.* **i**, 148.

